# Be Cautious with Crystal Structures of Membrane Proteins or Complexes Prepared in Detergents

**DOI:** 10.3390/cryst10020086

**Published:** 2020-02-03

**Authors:** Youzhong Guo

**Affiliations:** Department of Medicinal Chemistry & Institute for Structural Biology, Drug Discovery and Development, Virginia Commonwealth University, Richmond, VA 23298-0540, USA

**Keywords:** membrane protein, detergent, SMALP, native cell membrane nanoparticles system, AcrB, rhodopsin, TSPO, Energy-Coupling Factor type ABC transporter

## Abstract

Membrane proteins are an important class of macromolecules found in all living organisms and many of them serve as important drug targets. In order to understand their biological and biochemical functions and to exploit them for structure-based drug design, high-resolution and accurate structures of membrane proteins are needed, but are still rarely available, e.g., predominantly from X-ray crystallography, and more recently from single particle cryo-EM — an increasingly powerful tool for membrane protein structure determination. However, while protein-lipid interactions play crucial roles for the structural and functional integrity of membrane proteins, for historical reasons and due to technological limitations, until recently, the primary method for membrane protein crystallization has relied on detergents. Bicelle and lipid cubic phase (LCP) methods have also been used for membrane protein crystallization, but the first step requires detergent extraction of the protein from its native cell membrane. The resulting, crystal structures have been occasionally questioned, but such concerns were generally dismissed as accidents or ignored. However, even a hint of controversy indicates that methodological drawbacks in such structural research may exist. In the absence of caution, structures determined using these methods are often assumed to be correct, which has led to surprising hypotheses for their mechanisms of action. In this communication, several examples of structural studies on membrane proteins or complexes will be discussed: Resistance-Nodulation-Division (RND) family transporters, microbial rhodopsins, Tryptophan-rich Sensory Proteins (TSPO), and Energy-Coupling Factor (ECF) type ABC transporters. These analyses should focus the attention of membrane protein structural biologists on the potential problems in structure determination relying on detergent-based methods. Furthermore, careful examination of membrane proteins in their native cell environments by biochemical and biophysical techniques is warranted, and completely detergent-free systems for membrane protein research are crucially needed.

## 1. Introduction

Cellular membrane systems are crucial for all living organisms. They actively maintain the homeostasis of the cell and the body of a living organism. They are also responsible for sensing and reacting to environmental signals. Some of the most important biological reactions are conducted by membrane proteins; for example, photosynthesis is conducted by the photosynthetic reaction centers, rhythmic heartbeats depend on the coherent functions of membrane protein channels, and all activities of the nervous systems—from simple non-conscious reactions to human consciousness—depend on the function of numerous membrane proteins channels, transporters, enzymes, and receptors. Membrane proteins are the targets for more than 50% of modern pharmaceutical drugs [1].

Reviewing the short history of membrane protein structural biology, there are several important milestones: in 1975 the first structure of a membrane protein, bacteriorhodopsin, was determined at 7 Å by Henderson and Unwin using an electron microscope and 2D crystals [2]. This pioneering work eventually led to a Nobel Prize for Henderson in 2017. In 1985, Deisenhofer, Huber and Michel reported the first atomic-level resolution crystal structure of a photosynthetic reaction center prepared in detergent [3], which led to the 1988 Nobel Prize in Chemistry. In 2003, the Nobel Prize in chemistry was awarded to Agre and MacKinnon for their work on the structure and function of channels in cell membranes. In 2012, Lefkowitz and Kobilka were awarded the Nobel Prize for their studies of G-Protein-coupled receptors following the determination of the crystal structure of the β2 adrenergic receptor-G protein complex [4]. Because of the difficulties in crystallizing the TRPV1 channel, Julius, Cheng and colleagues used single particle cryo-EM to determine its first high-resolution structure [5]. This resolution-revolution was likely responsible for the Nobel Prize awarded to Dubochet, Frank and Henderson for their development of cryo-EM microscopy.

Each of these achievements was backed up by key technical developments, such as the invention of cryo-EM, application of detergents for membrane protein sample preparation, development of hetero-expression systems for membrane proteins, development of lipidic cubic phase (LCP) crystallization, and resolution improvements in direct detectors for single particle cryo-electron microscopy. Following the introduction of detergents for membrane protein sample preparation, and the first reported high-resolution crystal structure determination of a membrane protein complex by Deisenhofer and colleagues, as of November 12, 2019, 960 unique membrane protein structures have been determined according to the website “Membrane Proteins of Known 3D Structure” maintained by Stephen White’s Laboratory at UC Irvine. These structures have been mainly determined by X-ray crystallography. Crystallization of membrane proteins, especially human membrane proteins, is extremely difficult. The structure determination of nearly one thousand membrane proteins is an extraordinary achievement, but there is cause for concern: the vast majority of this data was obtained using methods for extraction and/or crystallization that rely on detergents. Unfortunately, there are significant drawbacks of detergent use in membrane protein structural biology.

Not all membrane proteins are amenable to detergent isolation and some proteins are only stable in specific detergents, while being completely unstable in others. Thus, a detergent screen is usually performed for each membrane protein to find the one that can best stabilize it and ideally keep it in its functional state. The reality is that for many membrane proteins it is difficult, and sometimes impossible, to find detergents that are stabilizing. Consequently, membrane protein structural biologists often have to take a detour and reengineer their protein of interest or find homologs from other species that can be stabilized and are suitable for crystallization or other structural studies. Protein engineering strategies include mutagenesis, truncation of loops or the N- or C-termini, insertion of fused proteins, or creating chimeric proteins by exchanging protein domains between structurally and functionally relevant proteins. One particularly famous strategy for crystallization of GPCRs is to fuse the T4 lysozyme to the third loop (IL3) [6]. All these strategies may provide useful structural information for understanding the membrane protein, but the drawbacks are also obvious in that the structures of the engineered or homologous proteins are different from the original. These structural differences, even if very subtle, may be crucial to understanding the mechanistic properties of the protein. In other words, misleading conclusions may be more readily drawn from the structural information provided by the homologous or engineered proteins.

Another difficulty in membrane protein structural biology arises from the complexity of protein-lipid interactions. The importance of the protein-lipid interactions cannot be overstated. Bulk lipids allow lateral movement of membrane proteins on the cell membrane, annular lipids form shells around the transmembrane domain and allow for exchange with the bulk lipids, non-annular lipids often remain tightly bound to membrane proteins as structural supports or active co-factors. For example, cholesterol has been known to affect the binding affinity of agonists for GPCRs and activation of Ca^2+^ ATPase is found to be diacylglycerol dependent [7,8]. Unfortunately, these detergent-based methods for membrane protein structural biology have significant and intrinsic drawbacks because they are based on the potentially flawed assumption that membrane proteins extracted from the lipid bilayer will be structurally and functionally the same, or at least similar to how they are on the cell membrane. This assumption relies on the bolder implicit assumption that the lipid bilayer is not an essential part of membrane proteins.

Realization of the limitations of detergent-based strategies for the crystallization of membrane proteins has led to other novel methods to crystallize membrane proteins within cell membrane lipid bilayer mimetics, e.g., lipid cubic phase (LCP) [9], bicelles [10], high-lipid-detergent concentration (HiLiDe) [11]. These methods have many advantages compared to crystallizing in detergent solutions. A landmark study using these methods was the determination of the high-resolution crystal structure of β2 adrenergic receptor G-protein complex in LCP [4]. More recently, single-particle cryo-EM has emerged as a powerful approach for structure determination of membrane proteins that are resistant to crystallization.

Regardless of whether or not the structural information obtained from membrane protein crystallography is generated with benign disregard for the importance of protein-lipid interactions, such data could be misleading. In this communication, my goal is to alert the readers of this widespread problem and offer supporting evidence with several analyses of previously published membrane protein structures where the protein-lipid interactions were clearly significant, and their absence affects our understanding of their inherent biology. These structural analyses will cover: (1) AcrB, the multidrug efflux transporter (2) Microbial rhodopsins (3) Tryptophan-rich sensory proteins (TSPO) and (4) ECF type ABC transporters.

## 2. Case Analysis

### 2.1. Structural Analysis of the Multiple Drug Efflux Transporter Protein AcrB: Interpretation of Crystal Structure of the Membrane Protein without Associated Lipids Could be Misleading

**Figure 1.**
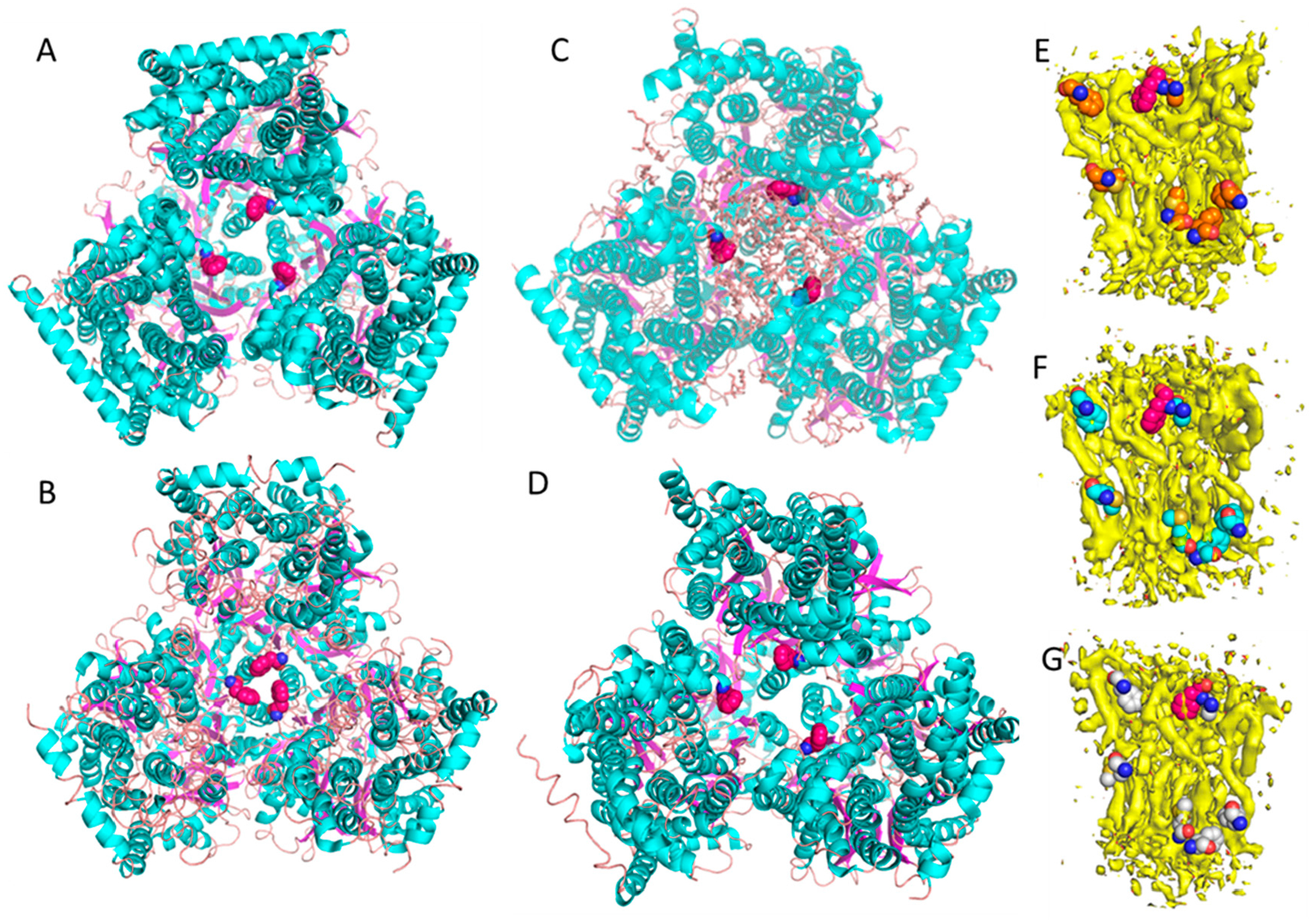
Crystal structures and cryo-EM structures of AcrB. **A**. Crystal structure of wild type AcrB with perfect three-fold symmetry. Sample was treated with detergent causing natural lipid molecules within the transmembrane domain to be washed out during sample preparation (PDB: 6BAJ). **B**. Crystal structure of AcrB D407A mutant (PDB: 6CSX). Sample was prepared with detergents. Once the mutation was made, the crystal structure showed a dramatically different conformation, indicated by the significantly smaller distances between the three-fold symmetrical F386 residues from 16 Å (Figure 1A) to 6 Å (Figure 1B). **C**. Single particle cryo-EM structure of wild type AcrB solved using the native cell membrane nanoparticle system that preserved the native lipid bilayer within the transmembrane domain (the same structure was used in generating Figures E, F, and G from each of the three sides of the asymmetric trimeric subunits). **D**. Single particle cryo-EM structure of AcrBD407 mutant prepared with NCMNS. The inter-F386 distances within the wild type AcrB and the mutant AcrB show no observable differences. **E**. F386 side chain from AcrB subunit A tightly associated with the lipid bilayer patch. **F**. F386 side chain from AcrB subunit B tightly associated with the lipid bilayer patch. **G**. F386 side chain from AcrB subunit C tightly associated with the lipid bilayer patch. Note: Phe386 is displayed as red colored sphere.

The multidrug efflux transport, AcrB is part of the AcrA-AcrB-AcrZ-TolC drug efflux protein complex in *E.coli*. AcrB belongs to the RND superfamily and functions as a trimer [12]. This transporter uses proton motive force to export exotic substances that could be harmful to *E. coli*. AcrB serves as a model membrane protein for method development in membrane protein structural biology mainly because it has an appropriate molecular size for single particles cryo-EM analysis and it has a known lipid bilayer patch within the transmembrane domain, which is susceptible to detergents. It is also a notorious contamination protein on nickel affinity columns [13]. Since 2002, numerous crystal structures of AcrB have been reported and all of them utilized detergent-based purification schemes. Most of these structures are in symmetric trimeric states, while a few have displayed asymmetric states [12,14–19]. In all of the crystal structures, the transmembrane domain contains a large lipid cavity, but because detergents were used to extract AcrB from its native cell membrane, the associated lipid bilayer patch had always been washed out. The transmembrane domain of AcrB contains several conserved residues, such as D407, D408, K940, and T978, and these residues have been known to be functionally important for the transport activity [20]. Traditionally, in order to investigate the active mechanism, the conserved residues were mutated and the crystal structure of the mutant was determined and compared with that of the wild type. Following this traditional analysis strategy, Edward Yu and colleagues made several mutants of AcrB with each of the conserved residues listed above. They found that each of these mutant structures were very similar to each other, but all were dramatically different from the crystal structure of the wild type AcrB [20]. For example, the distances between the three F386 residues (one for each subunit of the trimer), changed from 16 Å (Figure 1A) to 6 Å (Figure 1B) [20]. This dramatic conformational change was believed to be important and was featured in a proposed transport mechanism for the pump. However, since lipid components are often crucial for structural stability and the normal functioning of membrane proteins, we thought that structurally characterizing AcrB in a more native environment would be revealing.

We have been developing a detergent-free Native Cell Membrane Nanoparticles System (NCMNS) for membrane protein structural biology. In a test of this, we determined the cryo-EM structure of wild type of AcrB at 3.2 Å (Figure 1C) [21]. Surprisingly, we found a patch of the native cell membrane lipid bilayer within the lipid cavity of the transmembrane domain and a lipid belt wrapped around the transmembrane domain. Because the lipid patch within the transmembrane domain was tightly packed, we hypothesized that the dramatic conformational change indicated by the X-ray crystal structures should be impossible, due to steric effects caused by the presence of the lipids. Thus, we determined the AcrB D407A cryo-EM structure at 3.0 Å (Figure 1D) [21], which possessed a similar lipid bilayer patch within the transmembrane domain. Reviewing the relative positions for the three F386 residues, on the three AcrB subunits, showed that F386 is supported by the lipid bilayer. There was very little difference between the wild type AcrB and mutant AcrB D407 structures in this respect (Figure 1C,D). The cryo-EM-based observations, where the proteins are in more native conformations, are distinctly different from the results obtained with the X-ray crystal structures of AcrB, where the detergent *n*-dodecyl-β-D-maltopyranoside (DDM) was used in extraction and crystallization. We also found that the asymmetric trimer has a different conformation with respect to both the symmetric trimer and asymmetric trimer crystal structures. This is because the lipid bilayer buttresses the trimeric transmembrane domain. With careful examination of the EM density, we found that each of the F386 residue side chains tightly associate with the lipid bilayer within the transmembrane domain (Figure 1E–G).

This case study clearly proves that lipids are important for the structural and functional integrity of membrane proteins, and membrane protein crystal structures determined with detergent-based sample preparation need to be treated with all due caution.

### 2.2. Structural Analysis of Microbial Rhodopsins and Tspo Proteins: Native Oligomeric States of Membrane Proteins May not Be Preserved after Detergent Extraction or Reassembly Even in the Best Cell Membrane Mimetic Systems

Oligomerization is often crucial for the structural stability and normal physiological function of many membrane proteins and microbial rhodopsins have been known to exist as oligomers. Bacteriorhdopsin is a protein used by Archaea to capture light and use it as an energy source for pumping protons across the membrane and out of the cell. The resulting proton gradient is subsequently converted into chemical energy. As one of the first structurally characterized membrane proteins, bacteriorhodopsin has been a model protein for the field of membrane protein structural biology and method development. Lipid molecules and the lipid bilayer environment play important roles in maintaining the natural oligomeric states of membrane proteins. Henderson’s work with bacteriorhodopsin pioneered structure determinations of membrane proteins with cryo-EM and, accordingly, received the 2017 Nobel Prize. In their 2D crystals, bacteriorhodopsin existed as a trimer [2].

**Figure 2.**
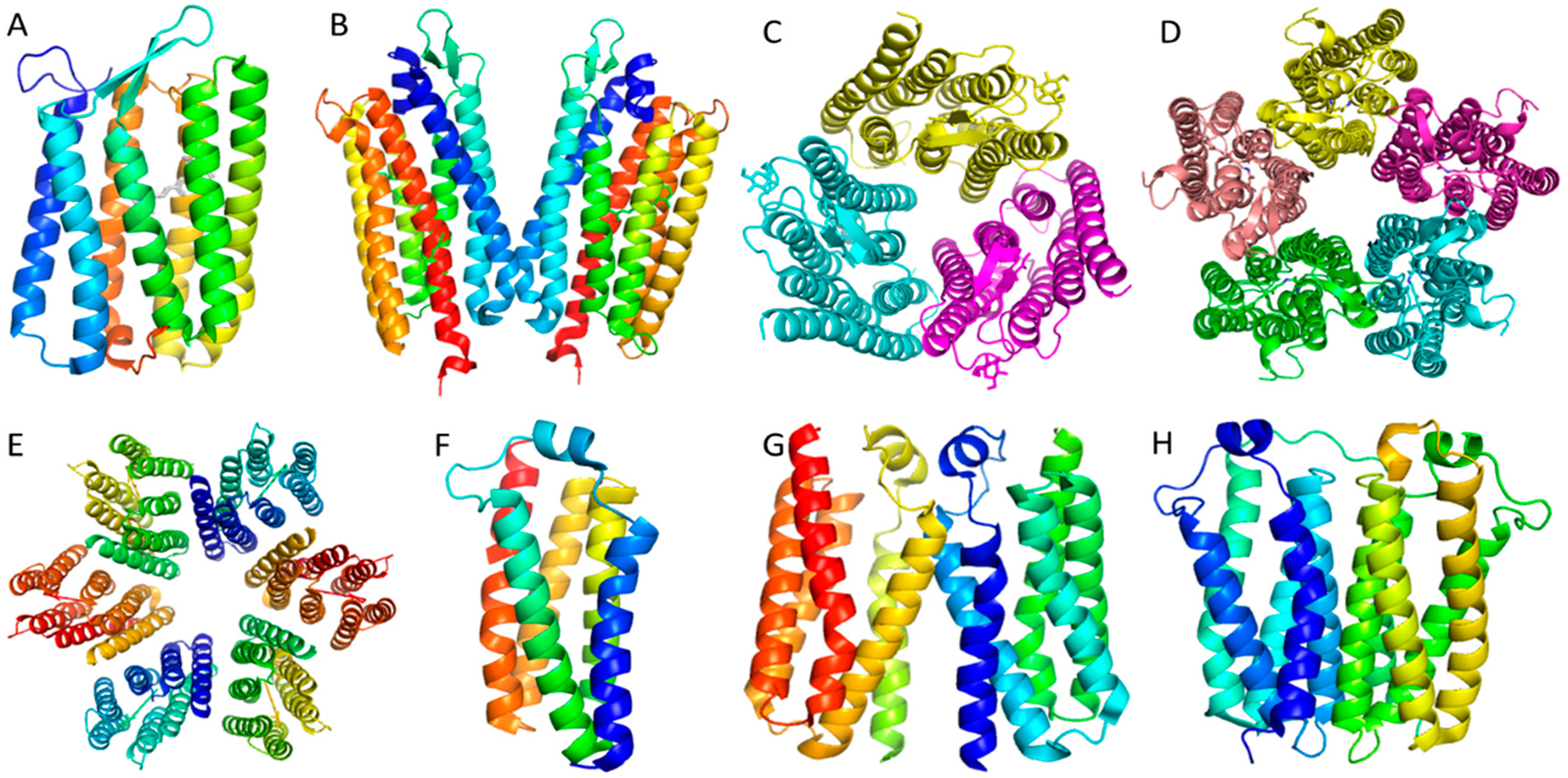
Controversial oligomers of membrane proteins crystallized in different conditions. (**A**). Bacteriorhodopsin monomer in the bicelle crystal. (**B**). Rhodopsin dimer in LCP crystals. (**C**). Rhodopsin trimer in LCP crystals. (**D**). Rhodopsin pentamer in detergent crystals. (**E**). Rhodopsin hexamer in LCP crystals. (**F**). *Bacillius cereus* TSPO monomer in LCP crystals. (**G**). *Bacillus cereus* TSPO dimer in LCP crystals. (**H**). Rhoder bacterial TSPO dimer in LCP crystals.

However, the reported 3D crystal structures show quite conflicting results: Faham and Bowie reported that bacteriorhodopsin existed as monomer in crystals obtained from bicelles [10]. Morael and colleagues also determined an LCP crystal structure of rhodopsin as a monomer (Figure 2A, PDB: 6GUY, unpublished data). Niemann and colleagues crystallized bacteriorhodopsin in detergent as a trimer (Figure 2C, PDB: 5AHZ) [22]. Girdeliy and colleagues also reported a trimeric LCP crystal structure of the bacteriohodopsin similar to the trimer in detergent [23]. Nureki and colleagues reported a bacterial rhodopsin sodium pump as dimers in LCP crystals (Figure 2B, PDB: 3X3C) [24]; while Gordeliy and colleagues reported the same sodium pump as pentamers in LCP crystals (Figure 2D, PDB: 4XTN) [25]. Lastly, Luecke and colleagues reported that proteorhodopsin existed as a hexamer in crystals grown in detergent solution (Figure 2E, PDB: 4JQ6) [26]. Table 1 summarizes the source organisms, expression organisms and the detergents used for crystallization of the microbial rhodopsins mentioned above. Clearly, microbial rhodopsin oligomeric states are controversial with a wide range of documented structures. What is the real natural oligomeric state? If it is a trimer, then the monomeric crystal structure is an artifact, as is the dimer structure. If the native oligomeric state is a pentamer, then the hexamer oligomer could be an artifact. One explanation for this confusion is that some of the oligomeric states observed in the crystal structures are non-native because of the loss of the membrane lipid environment that is responsible for supporting the rhodopsins’ structure and function. In an effort to solve the controversy over microbial rhodopsin’s oligomeric state, Uchihashi and colleagues used atomic force microscope (AFM) to image the microbial rhodopsins and they found that the Krokinobacterial rhodopsin sodium pump existed as pentamers. They also found the proton-pumping Green Proteorhodopsin (GPR) existed as a mixture of pentamers and hexamers (Figure 2D,E) [27]. It is also notable that AFM could not be ideally used in these studies if detergents were used to extract the membrane proteins.

Membrane bilayer mimicking systems such as bicelles, LCP and HiLiDe were developed to circumvent the major bottlenecks of current membrane protein crystallization methods. With bicelles, once the membrane protein is initially isolated with detergent by removing the natural lipids that interact with the oligomer, the protein is reconstituted into the artificial bicelle lipids. Here the protein may reform into an oligomeric state different from the native state. LCP is the most successful membrane mimetic system for crystallization of many GPCRs and other small membrane proteins. HiLiDe preserves the advantages of classical lipid-based methods and is compatible with both vapor diffusion and batch crystallization techniques. However, all membrane mimetic systems have a common disadvantage, which is that they do not necessarily provide physiological lipid environments. Hence, none can be completely trusted to maintain or rescue the natural oligomeric states of membrane proteins, because once detergents have been used for extraction of the proteins, the detergents may remove lipids that interact with the native protein structure that are crucial for maintaining the natural oligomeric state. As noted above, both the bicelle and LCP methods have yielded microbial rhodopsin structures as monomers, dimers, trimers and pentamers. Similarly, when the HiLide method was used to crystallize Ca^2+^ dependent P type ATPase LMCA1 from *Listeria monocytogenes*, it was found to be monomeric [28], likely because of the involvement of detergent in preparing these samples. However, our EM analysis with native cell membrane nanoparticles suggests that it could exist as dimers (unpublished data).

Microbial rhodopsins and their oligomeric states may actually be the leading indicator of widespread problems in detergent-based membrane protein crystallography. We have also studied the Tryptophan-rich Sensory Protein, (TSPO) a conserved membrane protein in terms of both structure and function, found in species ranging from bacteria to plants, animals, and humans [29]. Structures of TSPO had been previously investigated by NMR, X-ray crystallography, and cryo-EM [30]. Because the EM structure is about 10 Å in resolution, it provides little structural information. Interestingly, the NMR structure of mouse TSPO is dramatically different from that of bacterial TSPO, although both bind PK11195, a ligand specific for TSPO [29]. Considering the conservation of structure and function, one would expect to see high similarity between the X-ray structure of the bacterial TSPO in complex with PK11195 and NMR structure of mouse TSPO also in complex with PK11195. However, they are completely different in their binding modes and even the binding pockets are dramatically different [29,31]. These observations raised a natural concern: why are these structures so different? A second bacterial TSPO crystal structure has been recently reported and comparison between the two bacterial TSPO structures revealed high similarity, but despite the high similarity, these crystal structures displayed different dimeric conformations (Figure 2G,H) and TSPO in LCP has been reported to also exist as a monomer (Figure 2F) [29,32]. Thus, the real oligomeric state of TSPO remains unknown.

The intrinsic limitations of detergents in maintaining the natural and physiological oligomeric states of membrane proteins highlight the need for true detergent-free methods for extracting and structurally characterizing membrane proteins. If even large-scale effects like producing crystals with the correct oligomeric states are suspected to be incorrect, how reliable are the finer details of structure? There is significant promise, however, in the rapidly developing technology of native cell membrane nanoparticles systems, which we believe will be the right tool for investigating the natural oligomeric states of membrane proteins.

### 2.3. Structures of Ecf Transporter Complexes: the Crystal Structures of Membrane Proteins Prepared with Detergent May not Be Biologically Relevant and Mechanisms of Action Proposed Thereof May not be Correct

**Figure 3.**
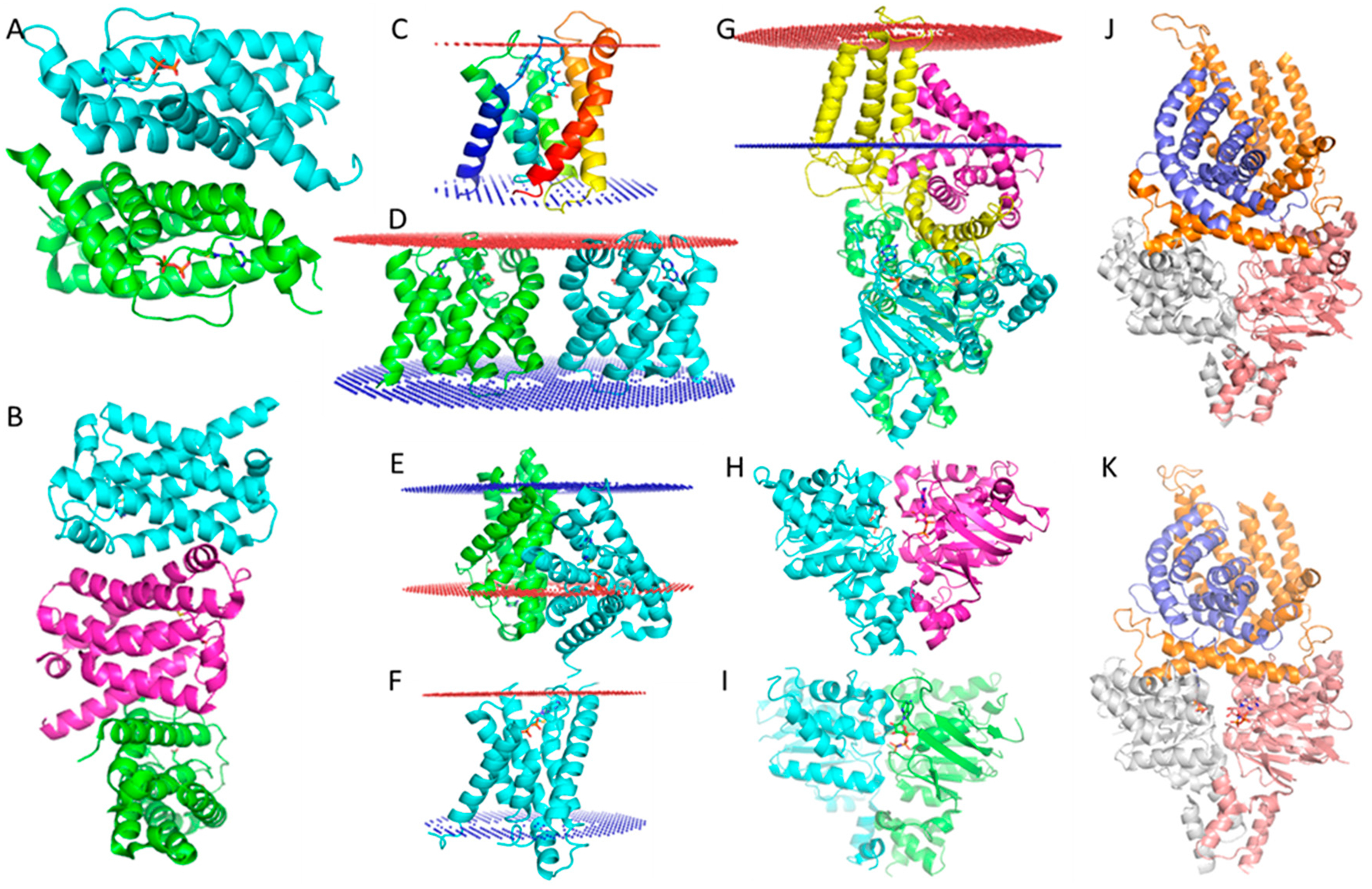
Crystal structures of ECF type of ABC transporter. (**A**). Crystal structure of the S component of the ECF type ThiT transporter. In the asymmetric unit, two monomers formed an artificial dimer caused by the lipid environment being damaged by detergents. (**B**). Crystal structure of the ECF type Folate transporter S component. Three S subunits associated into an artificial trimer through two very different dimeric interfaces because of detergent damage to the lipid environment. (**C**). Transmembrane region analysis with PPM of one S component of the folate transporter. (**D**). Transmembrane region analysis with PPM of an artificial S component dimer of the folate transporter. The relative orientation of the same S component when analyzed as a monomer is dramatically different from the analysis of the artificial dimer; in the artificial dimer the St component is tilted about 45°. (**E**). Transmembrane region analysis of ThiT artificial dimer. In this analysis, one S subunit has a normal orientation, however, the other subunit is almost parallel to the cell membrane and strikingly almost half of the S component is outside of the cell membrane. (**F**). Transmembrane region analysis of a single S component of ThiT, displaying one parallel S component in the same orientation as seen in Figure 3E; however, in the single S component analysis, we see the S component displaying a normal orientation. (**G**). PPM analysis of the transmembrane region of an ECF type ABC transporter. The T component is in a normal orientation within the calculated transmembrane region; however, the S component is parallel to the transmembrane region. This situation is strikingly similar to the situation displayed in Figure E. Since the dimer in Figure E is an artifact caused by detergent, the evidence suggests the crystallographic structure of the ECF transporter complex is an artifact. (**H**). ATPase dimer in an open state conformation without ATP or AMPPNP binding. (**I**). ATPase dimer in a closed state conformation with AMPPNP binding. Binding of AMPPNP induces the closed state conformation of the dimer. (**J**). ECF transporter complex *apo* state without ATP or AMPPNP binding to the ATPase domain, ATPase is in an open state conformation. (**K**). ECF transporter complexed with AMPPNP binding to the ATPase domain, however, the ATPase is still in an open state conformation. This is controversial since the ATPase binding to AMPPNP should induce a closed state conformation. The reason could be that the artificial complex is structurally and functionally aberrant, so AMPPNP could not induce the expected conformational change.

The ECF type of ABC transporters are a large class of non-canonical ABC transporters that contain four components, the S and T subunits, and two ATPase domains that form either a homo or heterodimer. The S and T components are transmembrane proteins [33]. Crystal structures of several ECF type ABC transporters have been reported: the S and T components are associated in an unusual relative orientation, where the T component is located on the cell membrane with helices vertical to the lipid bilayer, but the S component is almost parallel to the cell membrane bilayer [34,35]. This is rarely found in nature because transmembrane proteins are generally oriented more or less vertical to the cell membrane to maintain their free energy minimum. Transmembrane region analysis using the PPM server [36] predicts that the S component should be located on the cell membrane in an almost vertical orientation. In stark contrast to this prediction, the crystal structures of ECF Type ABC transporters display the helices as being almost parallel to the cell membrane and half of the S component as being outside of the cell membrane (Figure 3G) (PDB: 5D3M) [34,35,37]. Several crystal structures of ECF revealed that the S components can associate together in different dimeric interaction poses according to PDBePISA analysis [38], as shown in Figure 3A (PDB: 4TKR) [39] and 3B (PDB: 4DVE) [40]. While using the PPM server indicated that the S component monomer has a normal transmembrane region (Figure C, PDB: 4DVE), using a putative dimer in the transmembrane region analysis, produced dramatically different results (Figure 3D, PDB: 4DVE; Figure 3E, PDB: 4TKR). We know that the putative dimer is an artifact caused by detergent purification and is not biologically relevant.

Analysis of a putative thiamine transporter dimer using the PPM server reveals a very similar situation (Figure 3E, PDB:4TKR): one subunit (green) Is almost vertical to the cell membrane; however, the other monomer in parallel to the cell membrane and also halt the S component is again found outside of the cell membrane. This is strikingly similar to the orientations of the S and T components of the ECF transporter complex (Figure 3G, PDB: 5D3M). Again, we know that the putative dimer of the ECF transporter is an artifact caused by detergent purification and it is not biologically relevant. So, the unusual orientations of S and T in the thiamine complex are likely to also be artifacts caused by sample preparation with detergents.

Furthermore, it is well known that the dimeric ATPase can exist in two different states: (1) without nucleotide binding or with ADP binding where it exists in an open configuration (Figure 3H) (PDB: 4HLU) [41]; (2) with ATP or an ATP analog (AMPPNP) binding to the protein, where it exists in a closed state (Figure 3I) (PDB: 4ZIR) [42]. Since the conformational change of the ECF type ABC transporter in the transport cycle is driven by ATP, ATP binding or AMPPNP binding should lead to it being in the closed state. However, an AMPPNP complex crystal structure, as shown in Figure 3K (PDB: 5D3M), unexpectedly exists in an open state exactly like that of the *apo* state ECF transporter (Figure 3J, PDB: 5JSZ) [35]. This suggests the reported structure state may not be functional. All the previously mentioned observations have been regarded as normal with the presumption that the crystal structures of the ECF-transporter complex are all correct; however, in light of the potential problems caused by detergents, the evidence suggests these ECF type ABC transporter complex crystal structures are likely to be conformational artifacts. Therefore, the question that must be asked is: are these crystal structures really biologically relevant? The structural information needs to be rigorously examined by various biochemical and biophysical analyses before mechanisms of action are proposed.

## 3. Conclusions

Membrane proteins are naturally located on the cell membrane, but often we need to isolate the membrane proteins from their cell membrane for biochemical and biophysical analysis. While it has long been believed that the cell membrane plays an important role in maintaining the integrity of membrane proteins, in order to isolate these proteins from the cell membrane, we often have to use detergents. These detergents often lead to over delipidation of the protein, and lipids are crucial to the structural and functional integrity of the protein; their loss deprives the protein of structural stability and may corrupt its normal function. This is evident by how extracted membrane proteins are often unstable and/or nonfunctional. Despite this major compromise, the application of detergents has led to many successful advancements in structural biology. Likewise, it has led to many controversies.

Both detergents and artificial lipid molecules can damage membrane protein complexes; therefore, detergent-free and artificial lipid-free systems are in demand. For example, the entire plant metabolon complex is dynamic; while detergents failed to extract it, a membrane active polymer was able to catch the whole complex in native-like nanoparticles [43]. The native cell membrane nanoparticles system has also been successfully used to determine structures of lipid bilayers within a membrane transporter and can be used to determine the native oligomeric states of membrane protein complexes. One example of a detergent-free but not artificial lipid-free preparation is bacteriorhodopsin crystallized in LCP. While this crystal structure shows a trimeric state [44]; no native lipid was observed in the structure. Here, native lipids were substituted by monoolein. In our experience monoolein shocks and denatures the membrane protein due to the quick exchange of the solution with monoolein molecules. After the initial shock, the membrane protein refolds within the monoolein lipid environment, but because monoolein is a structurally different molecule than the substituted native lipids, disruption of the natural oligomer for rhodopsin is plausible. This suggests that not only detergents but also artificial lipids can lead to non-natural structures of membrane proteins.

A rigorous analysis of the problems caused by detergents in the process of structure determination for membrane proteins with NMR has recently been made [7]. Here, a few examples were selected to show that there are also potential problems caused by detergents in membrane protein crystallography. Similar problems in cryo-EM structure determination using detergents also exist, but are beyond the scope of this communication. The various structural problems and uncertainties seen in membrane proteins are not from the limitations of NMR, X-ray crystallography or single particle cryo-EM techniques as some have believed. The problems likely come from sample preparation.

Compared to soluble proteins, membrane proteins are unique in that the native structure and function of the protein is heavily dependent on their native lipid environment. The basic functional unit is not the membrane protein by itself, but rather the membrane protein and its associated lipid bilayer; this concept was recently was coined as “memtein” by Michael Overduin [45]. In principle, to investigate the structure and function of membrane proteins, the membrane protein should be kept in its native lipid environment. However, due to the high cost of protein production in human cell lines, and their slow cell growth, many researchers opt for alternative means of protein production. A common method used as a supplement for human cell expression systems is to express recombinant human proteins in *E. coli* cells; however, success rates of these efforts has been low for membrane proteins. One of the common problems with this method is the necessity for human membrane proteins to associate with cholesterol. This problem arises from the fact that the *E.coli* cell membrane does not contain cholesterol. While *E. coli* has been utilized as a successful expression system for many other exogenous proteins, the lack of a native lipid environment has greatly thwarted such efforts for membrane proteins. Recently the technology of cell-free expression has been developed to express difficult membrane proteins without using cells. The intrinsic limitation of this technology is neglecting the complexity of not only the native lipid environment, but the entire cellular system as a whole. Furthermore, even though broad application expression systems, such as *E.coli*, yeast or insect cells, have achieved many successful results for membrane protein structural biology, their use is not without concerns, especially when considering the importance of the native environment for the integrity of the memtein. In order to maintain the structural and functional integrity of membrane proteins, detergent-free systems should be utilized. Currently, SMALP and NCMNS are in development. Both SMALP and NCMNS use membrane active polymers to solubilize the cell membrane in the form of nanoparticles. The main limitation of SMALP is that the Styrene Maleic Acid polymers are not compatible with divalent ions and lower pH conditions. NCMNS was inspired by the SMALP system. However, unlike SMALP, NCMNS has a large membrane active polymer library that is compatible with divalent ions and much broader pH conditions. In this system, membrane proteins together with their associated annular and non-annular lipids are extracted as a whole unit in the form of nanoparticles. This system aims to determine high-resolution structures of membrane proteins in their native cell membrane lipid bilayer environment. Currently, not all membrane protein structures determined are biologically relevant and thus may not be accurate, because they are likely missing some structurally significant associated lipids. This is true regardless of whether or not they were determined using NMR, X-ray crystallography, or single-particle cryo-EM. Fortunately, detergent-free systems such as SMALP and NCMNS are being actively developed [21,46]. With true detergent-free systems, the great power of NMR, X-ray crystallography, micro-ED, and cryo-EM will be demonstrated by achieving more accurate structures of membrane proteins in their native states.

## Figures and Tables

**Table 1.** Crystal structures of microbial rhodopsins

Protein/PDB ID	Source Organism	Expression Organism	Detergent	Oligomer	Crystal Environment
Bacteriorhodopsin/PDB: 1BRD	*Halobacterium halobium*	*Halobacterium halobium*	Octylglucoside, dodecyl trimethyl ammonium chloride	Trimer	2D crystal
Bacteriorhodopsin/PDB: 1KME	*Halobacterium salinarum*	*Halobacierium salinarum*	Octylglucoside	Monomer	Bicelle
Archaerhodopsin-3/PDB: 6GUY	*Halorubrum sodomense*	*Halorubrum sodomense*	Not published	Monomer	LCP
Halorhodopsin,/PDB: 5AHZ	*Halobacierium salinarum*	*Halobacierium salinarum*	*n*-Octyl-β-D-glucoside	Trimer	Detergent solution
*Krokinobacter eikastus* rhodopsin 2/PDB: 3X3C	*Dokdonia eikasta*	*Escherichia coli*	*n*-Dodecyl-β-D-maltopyranoside	Dimer	LCP
*krokinobacter eikastus* rhodopsin 2/PDB: 4XTN	*Dokdonia eikasta*	*Escherichia coli*	Triton X-100, n-Dodecyl-β-D-maltopyranoside	Pentamer	LCP
blue light-absorbing proteorhodopsin/PDB: 4JQ6	Uncultured bacterium	*Escherichia coli*	*n*-Decyl-D-maltoside	Hexamer	Bicelle
